# Supplementary material for: COVID-19 challenges to dentistry in the new pandemic epicenter: Brazil
Source: PLoS One. 2020 Nov 30;15(11):e0242251. doi: 10.1371/journal.pone.0242251 (PMC7703993; doi:10.1371/journal.pone.0242251)
Supplement: S1 Table — (DOCX) [file pone.0242251.s001.docx]

**S1 Table. Questionnaire (original language: Brazilian Portuguese)**

| Impact of COVID-19 on routine of dental care assistance  Dear dentist  The clinical treatment of patients during the pandemic can put dentists in situations that involve a high risk of COVID-19 contagion. Clinical routine adaptations are being undertaken based on an understanding that revisions of routine in Brazil can help to improve dental care assistance for professionals and patients.  You are invited to participate in this research survey. The objective of the study is to assess the impact of the pandemic on dental practices in Brazil. Your participation will involve completing a quick survey about your clinical routine. If you do not want to participate, please leave the page or simply do not finish the survey. If you are not a dentist or did not graduate from dental school yet, we ask you not to participate. If you already answered this survey, we thank you for your participation.  Your participation is voluntary; no identification is necessary. If you agree, at the end of the survey, you can provide your email address to participate in a new round of the same survey. In this case, we guarantee that your data will be treated confidentially and anonymously. There is no remuneration for your participation in this survey. The results will be published in scientific articles and social media posts.  Potential benefits of the research: identification of the pandemic impact on the practice of dentistry, and prospection of solutions to help improve clinical routines.  Potential research risks: possible discomfort answering any given question. In this case, the option “I'd rather not say’ will be available. The option “Does not apply” can be used for questions you think do not apply to your case.  If you agree to participate, this page will be your Informed Consent Form. To maintain a copy, print this page or save it as a PDF file. If you need any clarification about this research or want to access additional information, at any moment, please contact the researchers (information below). If you prefer, you can contact us before answering the survey. You can contact us after submitting your questionnaire if you want to withdraw your participation consent.  If you agree to participate, we ask that you answer the survey completely. The average response time is 7 (seven) minutes.  **YOUR PARTICIPATION IS VERY IMPORTANT!**  Institution and researchers  Graduate Program in Dentistry  Federal University of Pelotas  Rua Gonçalves Chaves 457  96015-560 Pelotas-RS  Phone: (53) 3260.2831  Prof. Rafael Moraes (principal investigator) – rafael.moraes@ufpel.edu.br  Prof. Flávio Demarco – flavio.demarco@ufpel.edu.br  Prof. Giana Lima – giana.lima@ufpel.edu.br  Prof. Marcos Correa – marcos.britto@ufpel.edu.br  Prof. Maximiliano Cenci – maximiliano.cenci@ufpel.edu.br  Prof. Otávio D’Avila – otaviopereiradavila@gmail.com  Prof. Tatiana Cenci – tatiana.cenci@ufpel.tche.br  Research Ethics Board information:  School of Medicine  Federal University of Pelotas  Phone: (53) 3301.1801  Do you agree to participate in the study voluntarily?  0 – No  1 – Yes | |
| --- | --- |
| **Section 1: Questions related to your professional profile** | |
| 1. What is your gender? | 0 – Male  1 – Female  2 – I’d rather not declare (IPND) |
| 1. How old are you? | I’d rather not declare  List 18 – 80 or + |
| 1. From which type of institution did you graduate in Dentistry? | 0 – I did not graduate in Dentistry  1 – Public  2 – Communitarian  3 – Private  4 – Autarchy  5 – I don’t know  6 – I’d rather not declare  Other: |
| 1. In which year did you graduate in Dentistry?   *Attention: if you are not a dentist or did not graduate yet, please do not answer this survey.* | I’d rather not declare  List of years from 2020 to 1960 or before |
| 1. In which type of service do you work mostly?   *Mark only one oval.* | 0 – Public health network  1 – Team dental office, private network  2 – Solo private office  3 – Professor  4 – Management  5 – Research  6 – I am studying exclusively at the moment  7 – I don’t know  8 – I’d rather not declare  88 – Does not apply  Other: |
| 1. Have you completed postgraduate education in Dentistry?   *Please select the highest degree.* | 0 - No  1 – No but I’m currently enrolled  2 – Yes, short-term courses  3 – Yes, residency or special advanced training  4 – Yes, Masters or PhD  5 – I’d rather not declare |
| 1. In which Brazilian state does your work mostly take place? | I’d rather not declare  List of acronyms for all states + federal district |
| 1. Before the pandemic, how many patients did you assist, in average, in a complete work week? | I do not work with clinics or does not apply  I don’t know  I’d rather not declare  List of number: 1 to 100 or + |
| **Section 2: Questions related to your professional practice during the pandemic** | |
| 1. How do you describe your current work status in the place you work most? | 0 – I’m working as usual  1 – I’m working, but less frequently  2 – I’m assisting emergencies only  3 – I’m not working because of the pandemic  4 – I’m not working due to other reasons  5 – I’d rather not declare  88 – Does not apply |
| 1. How do you rate the impact of the pandemic in your clinical routine in the place you work most? | 0 – There was no impact  1 – Low impact  2 – Intermediate impact  3 – High impact  4 – Very high impact  5 – I don’t know  6 – I’d rather not declare  88 – I do not have a clinical routine or does not apply |
| 1. In average, how many patients are you currently assisting? | I do not work with clinics or does not apply  I don’t know how to answer  I’d rather not declare  None, because of the pandemic  None, due to other reasons  List of numbers 1 – 100 or + |
| 1. The routine in the place you work most is being determined mainly: | 0 – By yourself  1 – By your employer  2 – By regulations of authorities (dental councils, sanitary agencies)  3 – I don’t know  88 – Does not apply |
| 1. Did you participate on the decision of how to develop professional activities during the pandemic in the place you work most? | 0 – I did not participate  1 – I was consulted only  2 – I had active participation  3 – I’d rather not declare  88 – Does not apply |
| 1. Have you received any training on COVID-19 preventive measures? | 0 – No  1 – Only general instructions or online training  2 – I have received practical training  3 – I’d rather not declare  88 – Does not apply |
| 1. Which preventive measures did you receive training on?   *Mark all that apply* | 0 – I did not receive any training  1 – Use of different personal protective equipment (PPE)  2 – Standard PPE insertion and removal sequence  3 – Correct disposal of PPE  4 – Preparation of the office before appointments  5 – Preparation of the office between appointments  6 – Preparation of the office after appointments  7 – Infection control in the workplace  8 – COVID-19 screening in patients  9 – I’d rather not declare  88 – I do not work with clinics or does not apply  Other: |
| 1. How prepared do you feel to assist patients with confirmed diagnosis of COVID-19? | 0 – I do not feel any prepared  1 – Poorly prepared  2 – Intermediately prepared  3 – Well prepared  4 – Very well prepared  5 – I don’t know  6 – I’d rather not declare  88 – Does not apply |
| 1. Which of the following personal protective equipment (PPE) are available for all appointments in the place you work most?   *Please select only those which are always available.* | 0 – Disposable surgical mask  1 – PFF2 (N95) mask  2 – Reusable fabric mask  3 – Disposable surgical cap  4 – Fabric surgical cap  5 – Disposable coat  6 – Reusable fabric coat  7 – Waterproof coat  8 – Protective goggles  9 – Face shield  10 – I don’t know  11 – I’d rather not declare  88 – Does not apply  Other: |
| 1. Currently, which type of mask are you most frequently wearing for assisting patients? | 0 – Disposable surgical mask  1 – Reusable fabric mask  2 – PFF2 (N95) mask  3 – Surgical mask over PFF2 (N95) mask  4 – Two disposable surgical masks  5 – PFF2 (N95) mask over surgical mask  6 – Surgical mask over reusable fabric mask  7 – I’d rather not declare  88 – Does not apply  Other: |
| 1. Have you assisted any patients via online appointment during the pandemic? | 0 – No, and I’m not willing to at the moment  1 – No, but I’m willing to  2 – Yes, and I rate the experience as positive in general  3 – Yes, but I rate the experience as negative in general  88 – Does not apply |
| **Section 3: Structure at your main workplace** | |
| 1. Was the structure of your main workplace adapted to allow patients’ treatment during the pandemic? | 0 – No  1 – Yes, the waiting room  2 – Yes, the office  3 – Yes, the entire work environment  4 – I don’t know  5 – I’d rather not declare  88 – Does not apply |
| 1. Did changes in your clinical routine due to COVID-19 result in additional costs in your main workplace? | 0 – No  1 – Yes, but treatment prices were not adjusted for patients  2 – Yes, and treatment prices were adjusted for patients  3 – I do not know  4 – I’d rather not declare  88 – Does not apply |
| 1. Did you (or your employer) follow official recommendations for adapting the clinical routine in your main workplace? | 0 – Recommendations were not followed  1 – National Health Surveillance Agency/Brazil  2 – Health Ministry/Brazil  3 – Federal Council of Dentistry/Brazil  4 – Centers for Disease Control and Prevention/USA  5 – American Dental Association/USA  6 – I don’t know  7 – I’d rather not declare  88 – Does not apply  Other: |
| 1. Which of the following sources for updated information on professional conduct toward the pandemic do you access? | 0 – I do not access information  1 – Dental councils of dental associations  2 – Universities or research centers websites  3 – Scientific literature  4 – Health websites/blogs  5 – Social network services  6 – Other dentists  7 – Other health professionals  8 – I don’t know  9 – I’d rather not declare  88 – Does not apply  Other: |
| 1. Do you perform any type of patient screening concerning to COVID-19 before appointments in your main workplace?   *Mark all that apply* | 0 – No, I am using normal anamnesis  1 – Face-to-face application of specific questionnaire for COVID-19  2 – Previous application of specific COVID-19 questionnaire via telephone, text message or similar  3 – Temperature check of patients in the office  4 – Request a temperature check before the patient arrives at the office  5 – Recommend mouthwashes with antimicrobials in the office  6 – Recommend mouthwashes with antimicrobials for the patient before arriving at the office  7 – I Prefer not to declare  88 – Does not apply  Other: |
| 1. Which disinfectants or antimicrobial agents are you adopting to decontaminate surfaces or the environment in your main workplace?   *Mark all that apply* | 0 – None  1 – 70% alcohol  2 – Ultraviolet light  3 – Ozone-based sterilizer  4 – Bleach  5 – Diluted sodium hypochlorite  6 – Undiluted sodium hypochlorite  7 – Lodopovidone  8 – Phenolic compounds  9 – Ammonium quaternaries (e.g. benzalkonium chloride)  10 – I don’t know  11 – I’d rather not declare  88 – Does Not Apply  Other: |
| 1. Are you or your or your employer allowing patients’ companions in your main workplace? | 0 – I’m not working  1 – No, companions are forbidden  2 – Only in the waiting room  3 – Only children and/or people with special needs  4 – Yes, normally  5 – I don’t know  6 – I’d rather not declare  88 – Does not apply |
| 1. Do you currently fear to contract COVID-19 at work? | 0 – No  1 – Yes, a little  2 – Yes  3 – Yes, a lot  4 – I don’t know  5 – I’d rather not declare  88 – Does not apply |
| 1. Have you assisted patients with confirmed diagnostics of COVID-19? | 0 – No  1 – Yes  2 – I don’t know  3 – I’d rather not declare  88 – Does not apply |
| 1. Have you suspected or tested yourself for COVID-19?? | 0 – No  1 – Suspect without test  2 – I tested negative for COVID-19  3 – My test was inconclusive for COVID-19  4 – I tested positive for COVID-19  5 – I’d rather not declare  88 – Does not apply |
| 1. Do you agree with social distancing measures adopted currently in your city? | 0 – Fully disagree  1 – Partially disagree  2 – Not agree or disagree  3 – Partially agree  4 – Fully Agree  5 – I don’t know  6 – I’d rather not declare |
| This study will have a follow up, longitudinal phase. If you agree, please leave your email address so you can receive our communication in the future. Your email address will not be disclosed in any way and your answers will not be associated to your email (researchers’ confidentiality commitment). | Your e-mail: |
| THANK YOU VERY MUCH FOR YOUR PARTICIPATION! | |
